# miR-138-5p suppresses autophagy in pancreatic cancer by targeting *SIRT1*

**DOI:** 10.18632/oncotarget.14360

**Published:** 2016-12-29

**Authors:** She Tian, Xingjun Guo, Chao Yu, Chengyi Sun, Jianxin Jiang

**Affiliations:** ^1^ Department of Hepatic-Biliary-Pancreatic Surgery, The Affiliated Hospital of Guizhou Medical University, Guiyang, China; ^2^ Department of Biliary-Pancreatic Surgery, Affiliated Tongji Hospital, Tongji Medical College, Huazhong University of Science and Technology, Wuhan, China; ^3^ Department of Hepatic-Biliary-Pancreatic Surgery, Renmin Hospital of Wuhan University, Wuhan, China; ^4^ Hubei Key Laboratory of Digestive System Disease

**Keywords:** pancreatic cancer, miR-138-5p, autophagy, SIRT1

## Abstract

The role of microRNA in the aberrant autophagy that occurs in pancreatic cancer remains controversial. Because hypoxia is known to induce autophagy, we screened for differentially expressed microRNAs using a miRNA microarray with pancreatic cancer cells cultured under normoxic and hypoxic conditions. We found that miR-138-5p was among the most downregulated miRNA in hypoxia-stimulated cells, and that overexpression of miR-138-5p substantially reduced expression of autophagy markers. In addition, western blot and immunofluorescence analyses and electron microscopy revealed that miR-138-5p inhibited autophagy in pancreatic cancer cells and blocked serum starvation-induced autophagic flux independently of the typical autophagic signaling pathway. miR-138-5p had no effect on ATG3, ATG5, or ATG7, three primary autophagy-associated genes. Instead, miR-138-5p specifically targeted the *SIRT1* 3′ untranslated region and suppressed autophagy by reducing the level of SIRT1, which acetylates FoxO1 and regulates autophagy via FoxO1/Rab7. SIRT1 or Rab7 knockdown blocked the SIRT1/FoxO1/Rab7 axis and suppressed autophagic inhibition by miR-138-5p. Finally, we found that miR-138-5p inhibited autophagy and tumor growth *in vivo*. These results indicate that miR-138-5p suppresses autophagy in pancreatic cancer by targeting *SIRT1*.

## INTRODUCTION

Pancreatic cancer is the sixth leading cause of cancer death in China [1]. Because its incidence and mortality rates are gradually increasing, it is projected that pancreatic cancer will soon become the second leading cause of cancer death. Most pancreatic cancer patients have poor prognosis, because of advanced disease and tumors that cannot be fully resected, and their 5-year survival rate is less than 5%.

Autophagy is the dynamic process by which unnecessary or dysfunctional cytosolic proteins and organelles are degraded in order to maintain cell homeostasis [2]. It is rapidly triggered by nutrient starvation, hypoxic stress, and various other stress conditions [3]. Dysfunction of the autophagy pathway has been linked to various human cancers, either enhancing or preventing tumorigenesis [4]. However, it has been reported that the crucial role of autophagy in cancer cells is to maintain tumor cell survival, especially in solid tumors [5]. Inhibition of autophagy genes in tumor cells has been shown to induce cell death [6, 7]. Furthermore, in pancreatic cancer cells, autophagy defects are associated with both a malignant phenotype and poor prognosis [8–10].

MicroRNAs (miRNAs) are endogenous, highly conserved, small non-coding RNAs, 19–23 nucleotides in length. miRNAs bind to the base of the 3′-untranslated region (3′-UTR) of their target mRNA and suppress gene expression by degradation or translational inhibition. Growing evidence has shown that miRNAs modulate many cellular biological processes [11]. Several studies have indicated that deregulation of miR-138 promotes the progression of tumorigenesis in human neoplasms, including glioblastoma, lung cancer, head and neck squamous cell carcinoma, malignant melanoma, cervical cancer, and gastrointestinal neoplasms [12–15]. Furthermore, overexpression of miR-138-5p inhibits the proliferation of pancreatic cancer cells [16]. We compared the miRNA expression profiles of three normoxia cultured samples and three hypoxia-cultured samples using miRNA microarray. We then focused on role and underlying mechanism of miRNAs in autophagy in pancreatic cancer.

## RESULTS

### miR-138-5p inhibits the autophagy flux in pancreatic cancer cells

**Figure 1:**
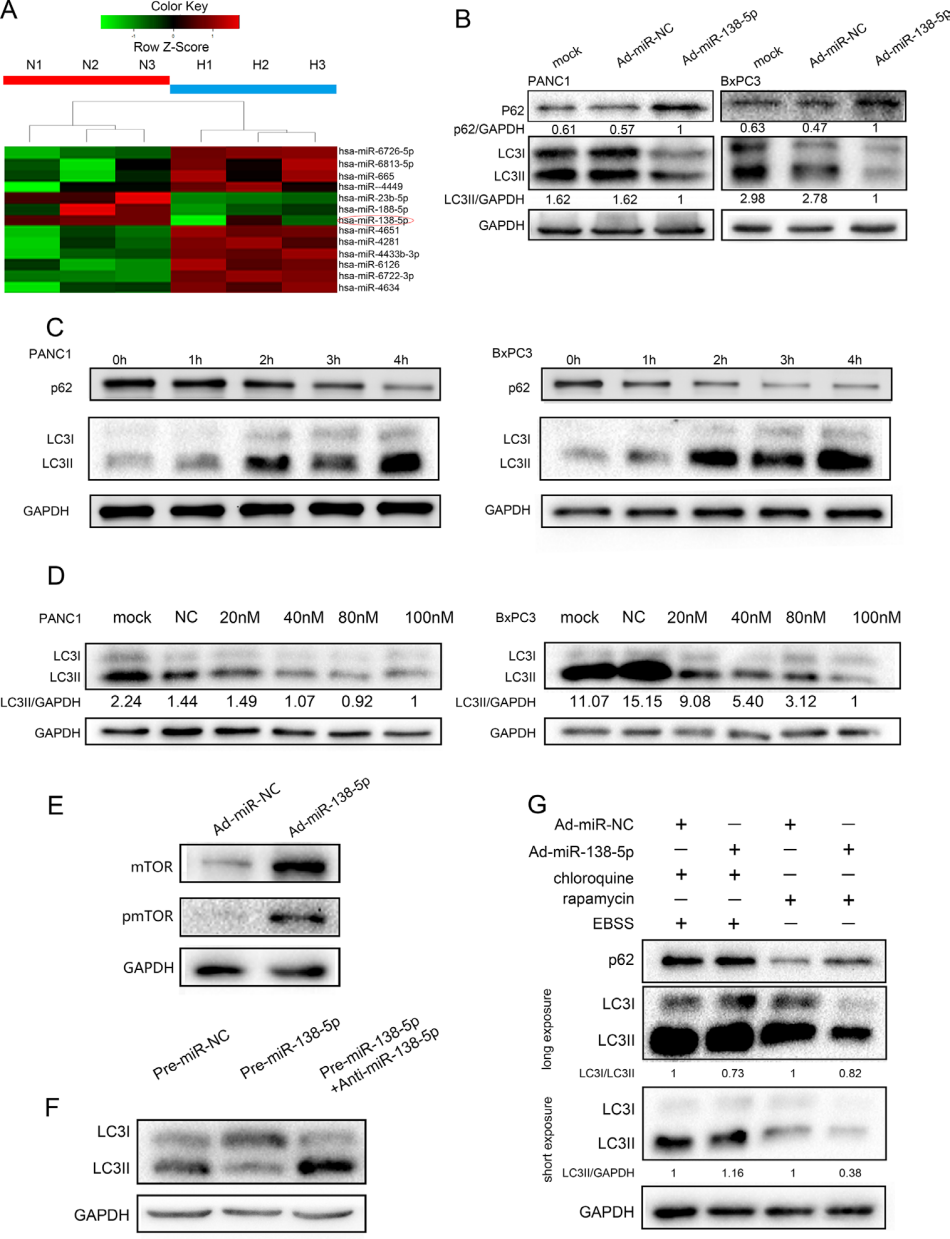
miR-138-5p inhibits the autophagy flux in pancreatic cancer cells (**A**) Differential microRNAs in normoxia or hypoxia cultured pancreatic cancer cells were screened. Heat map showing gene expression profiles. (**B)** pancreatic cancer cells transfected with Ad-miR-NC or Ad-miR-138-5p duplex were exposed to physiological hypoxia (1% oxygen) for 24 h and subjected to western blotting for LC3 and P62 expression (*n* = 3). (**C**) PANC-1 and BxPC-3 cells exposed to serum-free medium for the indicated time, were subjected to western blotting for LC3 and P62 expression (*n* = 3). (**D**) PANC-1 and BxPC-3 cells were transfected with the indicated doses of pre-miR-138-5p. LC3I/II protein was detected by western blotting (*n* = 3). (**E**) PANC-1 cells were not transfected, or transfected with NC or miR-138-5p duplex for 48 h. mTOR and phospho-mTOR were detected by western blotting after 8 h of exposure to serum-free medium (*n* = 3). (**F**) miR-138-5p precursor was transfected alone or with miR-138-5p inhibitor (anti-miR-138-5p) into PANC-1 cells. LC3I/II was detected by western blotting after 4 h of incubation in serum-free medium. Pre-miR-NC served as control (*n* = 3). (**G**) After transfection for 48 h, cells were treated with 10 μM chloroquine and/or rapamycin, and exposed to serum-free medium for 4 h. P62 and LC3I/II expression was detected by western blotting (*n* = 3).

To identify genes that are differentially expressed between pancreatic cancer cells cultured under normoxic and hypoxic conditions, we compared the miRNA expression profiles of three normoxia cultured samples and three hypoxia-cultured samples using an Agilent miRNA microarray. We found that 19 miRNAs were overexpressed and three were downregulated in the hypoxia group compared with the normoxia group (Figure 1A). To illustrate the function of miR-138-5p in autophagy, we generated stable miR-138-5p-overexpressing cells (miR-138-5pU), using an adenoviral delivery system. Ad-miR-NC was used as a control vector. The expression of miR-138-5p in these cells was verified by quantitative PCR (Supplementary Figure S1). Next, PANC-1 and BxPC-3 cells were exposed to physiological hypoxia (1% oxygen) and western blotting was performed to analyze the LC3I/II and P62 expression. Overexpression of miR-138-5p led to a decrease in LC3B-II expression and an increase in P62 protein expression in both PANC-1 and BxPC-3 cells (Figure 1B). When the cells were exposed to serum starvation medium, autophagy was clearly activated in PANC-1 and BxPC-3 cells, as reflected by the increased accumulation of LC3II and decreased P62 level (Figure 1C). Next, the effect of miR-138-5p on serum starvation-induced autophagy was investigated.

Overexpression of miR-138-5p by pre-miR-138-5p inhibited LC3II expression (Figure 1D). The expression of miR-138-5p in different groups were verified by quantitative PCR (Supplementary Figure S2). In addition, miR-138-5p overexpression inhibited mTOR expression and mTOR dephosphorylation (Figure 1E). Neutralization of miR-138-5p by its inhibitor, anti-miR-138-5p, restored autophagy activation, as indicated by increased LC3II expression (Figure 1F). We used chloroquine to inhibit autophagosome degradation in lysosomes after serum starvation. LC3II accumulation was increased in pre-miR-138-5p-transfected cells compared with the control cells (short exposure). However, when we used the mTOR inhibitor rapamycin, miR-138-5p suppressed LC3II expression and decreased the ratio of LC3II to LC3I after long exposure (Figure 1G).

**Figure 2:**
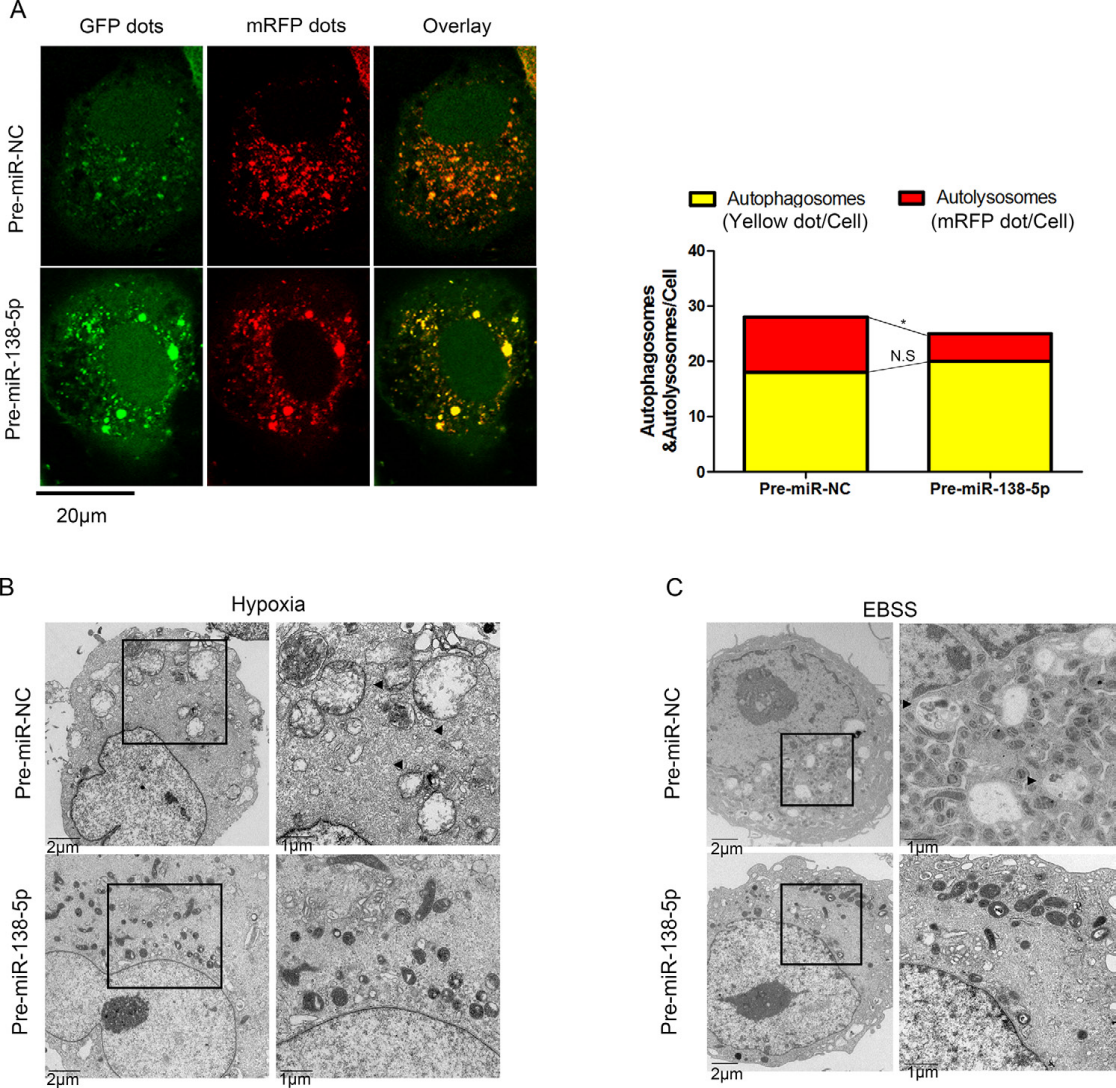
miR-138-5p inhibits the autophagy flux in pancreatic cancer cells (**A**) After transfection of PANC-1 cells with Ad-tf-LC3 for 72 h, the cells were transfected with pre-miR-NC or pre-miR-138-5p, and exposed to serum-free medium for 4 h. Representative images of fluorescent LC3 puncta are shown. The mean number of autophagosomes (yellow puncta in merged images) and autolysosomes (red puncta merged images) is shown in the right panel (*n* = 3). *P < 0.05. (**B**) Transmission electron microscopy showed autophagosomes (black arrow) in PANC-1 cells of the pre-miR-NC group after hypoxia for 48 h, whereas broken and swollen mitochondria were observed in the pre-miR-138-5p group without autophagic signs. (**C**) Transmission electron microscopy showed autophagosomes in PANC-1 cells of the pre-miR-NC group after serum-free medium culturing for 12 h. Mitochondria swelling and crista fragmentation were observed in the pre-miR-138-5p group (*n* = 3).

Next, we used an adenovirus harboring mRFP-GFP-LC3 (Ad-tf-LC3), to separately evaluate the autophagosome and autolysosome accumulation. miR-138-5p-overexpressing cells displayed fewer autolysosomes, indicated by free red puncta, compared with the NC group; however, autophagosomes, indicated by yellow puncta, were not significantly increased (Figure 2A). Furthermore, electron microscopy revealed many more autophagic vesicles in miR-NC-transfected cells compared with NC-138-5p-transfected cells upon hypoxia and serum starvation (Figure 2B, 2C). These results suggest that miR-138-5p blocked the autophagy flux.

### miR-138-5p blocks serum starvation-induced autophagy flux in a manner independent of the typical autophagy signaling pathway

**Figure 3:**
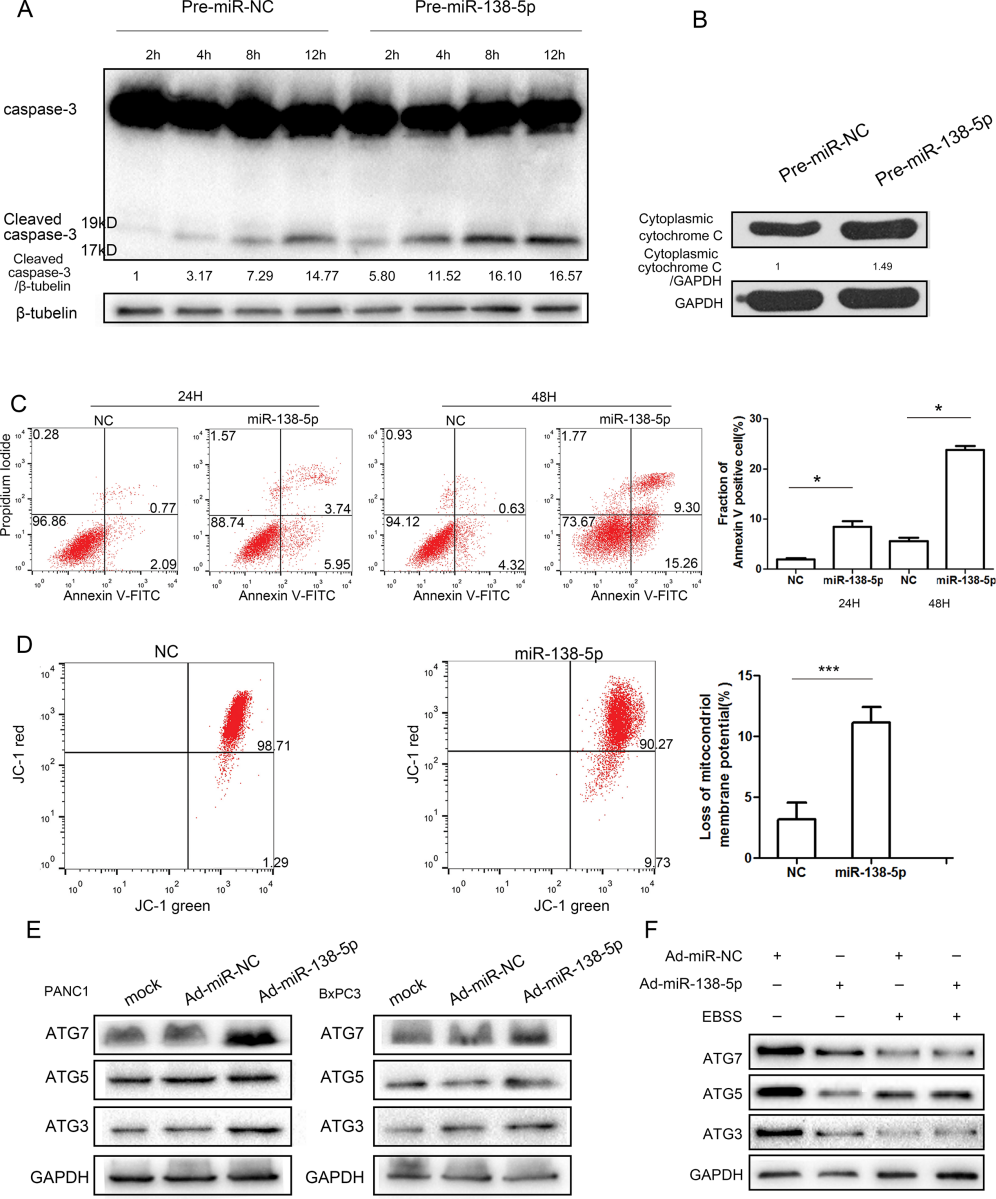
miR-138-5p promotes apoptosis and regulates autophagy in a manner independent of the ATG pathway (**A**, **B**) PANC-1 cells transfected with pre-miR-NC or pre-miR-138-5p were starved for the indicated time and subjected to western blotting, to examine the expression of caspase-3, cleaved caspase-3, and cytoplasmic cytochrome C (*n* = 3). PANC-1 cells were transfected with the indicated RNA duplex for 48 h, incubated in serum-free medium for another 24 h or 48 h, and then stained with annexin V-FITC/PI dual staining. The percentage of Annexin V-staining cells increased to 9.69 percent at 24 h and 24.56 precent at 48 h in the miR-138-5p-transfected group (**C**). (**D**) Mitochondrial membrane potential (MMP) was analyzed by flow cytometry after staining cells with JC-1 dye. The ratio of intensities of JC-1 monomers to JC-1 aggregates was analyzed and compared with values for control cells. The asterisks indicate an effect of treatment (**P < 0.01). After 48 h of transfection with pre-miR-138-5p or pre-miR-NC, cells were exposed to hypoxia for 24 h or to serum-free medium for 4 h. ATG3, ATG5 and ATG7 expression were examined by western blot analysis (**E**, **F**).

We hypothesized that miR-138-5p impaired serum starvation-induced autophagy by attenuating the protective effect of mitophagy. miR-138-5p upregulated active cleaved caspase-3 expression and cytoplasmic cytochrome C expression (Figure 3A, 3B). Overexpressing miR-138-5p restored the sensitivity to apoptosis (Figure 3C) and mitochondria in PANC-1 cells lost mitochondrial membrane potential (MMP) compared with control group. (Figure 3D). As miR-138-5p inhibited autophagosome formation and fusion, miR-138-5p's effect on the main autophagy-related proteins, ATG3, ATG5, and ATG7, was evaluated. However, miR-138-5p overexpression did not affect autophagy-associated protein expression (Figure 3E, 3F). Thus, we hypothesized that miR-138-5p inhibits mTOR dephosphorylation under serum starvation-induced autophagy by directly targeting other genes and regulating the autophagy flux.

### miR-138-5p suppresses autophagy by targeting SIRT1

**Figure 4:**
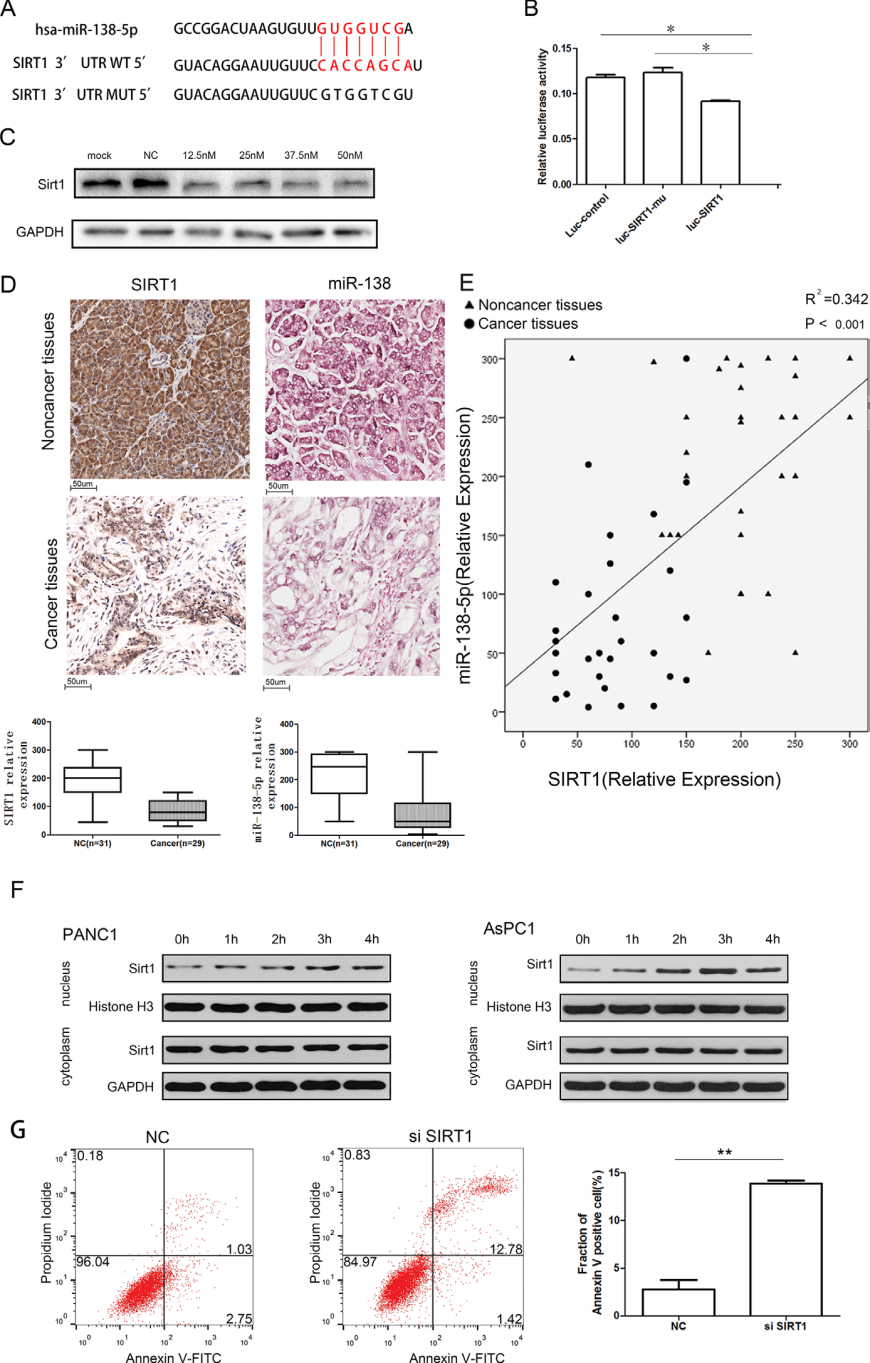
miR-138-5p inhibits autophagy by directly regulating the expression of SIRT1 (**A**) The predicted binding site of miR-138-5p in the 3′-UTR of *SIRT1* and the mutated 3′-UTR of *SIRT1* (SIRT1-mu) are shown. (**B**) PANC-1 cells were transfected with pmiR-REPORT control construct (Luc-control), mutant 3′-UTR *SIRT1* construct (Luc-FOXC1-mu), or wild type 3′-UTR *SIRT1* construct (Luc-SIRT1), along with β-galactosidase and lv-miR-138-5p constructs. Luciferase activity was detected 48 h after transfection. The firefly luciferase activity of each sample was normalized to the Renilla luciferase activity, (*P < 0.05, *n* = 6). (**C**) PANC-1 cells transfected with the indicated doses of pre-miR-138-5p were subjected to western blotting, to detect SIRT1 protein expression (*n* = 3). (**D**) Representative immunohistochemical staining of a tissue array containing pancreatic cancer samples with anti-SIRT1 antibody, and statistical analysis of the SIRT1 relative expression in adjacent noncancerous and tumor tissues. Representative *in situ* hybridization (ISH) staining on a tissue array, and the statistical analysis of miR-138-5p relative expression in adjacent noncancerous and tumor tissues. (**E**) Correlation analysis of SIRT1 expression and miR-138-5p in noncancerous and pancreatic cancer tissues. (**F**) PANC-1 and AsPC-1 were starved and subjected to western blotting as indicated (*n* = 3). (**G**) PANC-1 cells were nontransfected or transfected with the si-SIRT1 (100 nM) duplexes for 48 hours, then incubated in serum-free medium for another 24 hours, followed by staining with Annexin V-FITC/PI and flow cytometry analysis.

TargetScan analysis (TargetScan Human 6.0) revealed that *SIRT1* contains a classical and evolutionarily conserved miR-138-5p binding site in its 3′-UTR (Figure 4A). A luciferase reporter assay indicated that miR-138-5p significantly inhibited the luciferase activity of the reporter vector containing the wild type 3′-UTR of *SIRT1*, but not that of the empty vector or the mutant 3′-UTR vector, demonstrating specific targeting by miR-138-5p of the *SIRT1* 3′-UTR (Figure 4B). This effect was confirmed by western blotting of PANC-1 cells, as miR-138-5p inhibited SIRT1 expression when the indicated miRNA precursors were introduced (Figure 4C). Moreover, when examining noncancerous and pancreatic cancer tissues, SIRT1 was significantly overexpressed in the former specimens compared with the pancreatic cancer tissues (Supplementary Table S1). Additionally, *in situ* hybridization (ISH) demonstrated that miR-138-5p was downregulated in pancreatic cancer tissues (Figure 4D). Finally, we found that miR-138-5p expression positively correlated with SIRT1 (Figure 4E). Starvation-induced autophagy prompted SIRT1 translocation into the nucleus in a time-dependent manner (Figure 4F). Additionally, SIRT1 knockdown increased the fraction of Annexin V positive cells to 14.2% (Figure 4G).

### SIRT1 deacetylates FoxO1 and regulates autophagy via FoxO1/Rab7

**Figure 5:**
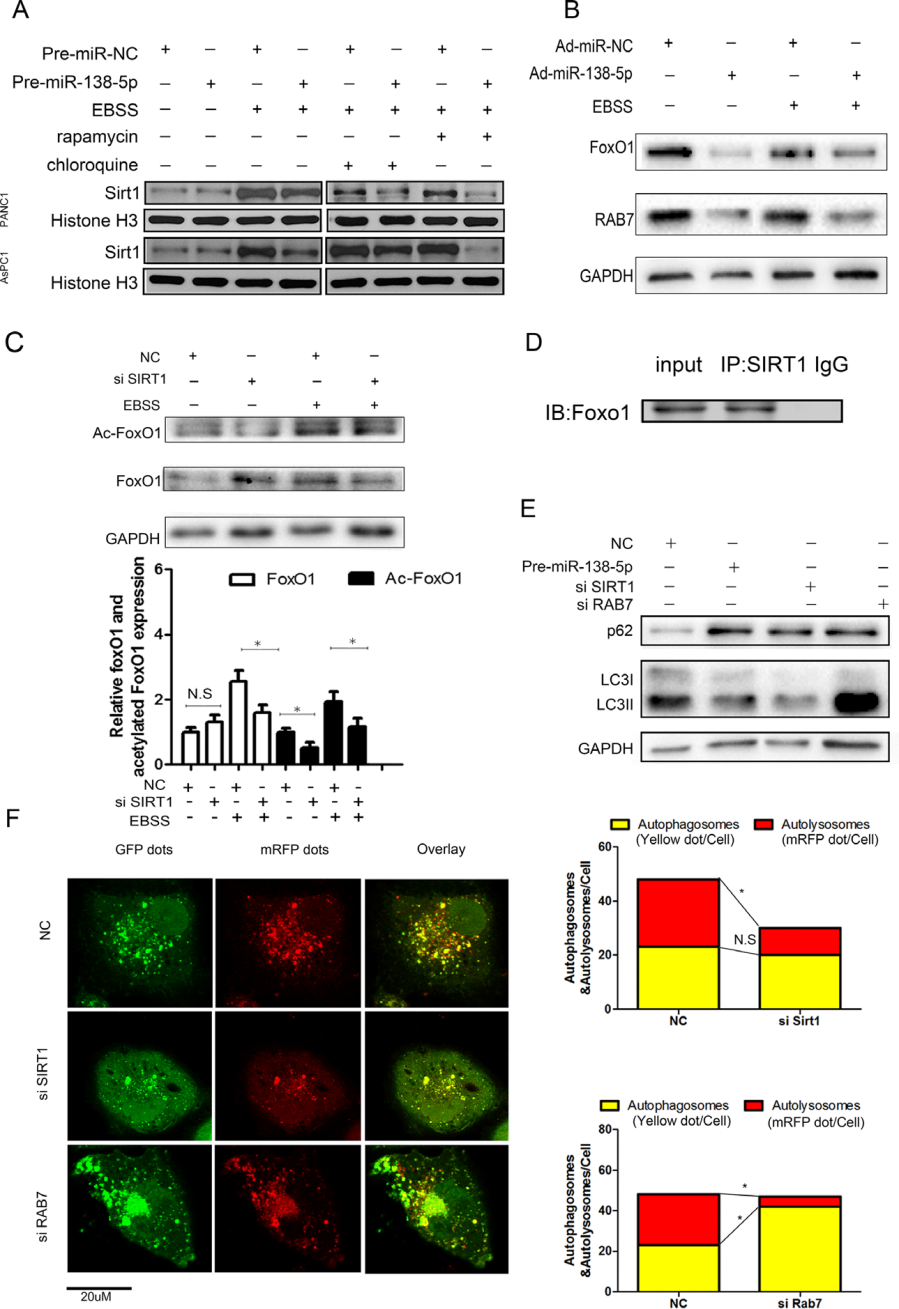
SIRT1 regulates autophagy via the FoxO1/Rab7 pathway (**A**) PANC-1 cells transfected with NC or miR-138-5p for 48 hours were subjected to western blot as indicated. (**B**) miR-138-5p precursor alone or miR-NC was transfected into PANC-1 cells, which were then starved for 8 h to induce autophagy. FoxO1 and Rab7 expression was examined by western blotting (*n* = 3). (**C**) PANC-1 cells transfected with NC or siSIRT1 for 48 h were incubated in serum-free medium for another 4 h, subjected to western blot analysis to detect acetylated FoxO1 and total FoxO1 expression. (**D**) Immunoblot analysis showing co-immunoprecipitation of endogenous SIRT1 and FoxO1. (**E**) PANC-1 cells transfected with pre-miR-138-5p, siSIRT1 or siRab7, and treated with serum-free medium for 4 h were subjected to western blot analysis as indicated. (**F**) PANC-1 cells were transfected with Ad-tf-LC3 for 72 h, and then with siNC, siSIRT1 or siRAB7. Representative images of fluorescent LC3 puncta are shown.

Autophagy increased the expression of SIRT1 in the nucleus, and miR-138-5p decreased the expression of SIRT1 in PANC-1 and AsPC cell lines (Figure 5A). miR-138-5p-overexpressing and negative control PANC-1 cells were stimulated by serum starvation and subjected to western blotting, in order to examine FoxO1 and Rab7 expression. miR-138-5p overexpression led to a decrease in FoxO1 and Rab7 levels (Figure 5B). To further confirm the effect of SIRT1 on autophagy, the possibility that SIRT1 regulates PANC-1 autophagy by acetylating FoxO1 was examined using PANC-1 cells transfected with *SIRT1* siRNA or NC siRNA for 48 h. Starving PANC-1 cells for 4h decreased the Ac-FoxO1 protein expression, compared with the control group (Figure 5C). Moreover, a co-immunoprecipitation assay revealed that SIRT1 interacts with FoxO1 in PANC-1 cells (Figure 5D). To further evaluate the role of the SIRT1/FoxO1/Rab7 axis in autophagy, PANC-1 cells transfected with *SIRT1* or *Rab7* siRNA were subjected to western blot analysis (Figure 5E). SIRT1 or Rab7 expression was verified by western bloting (Supplementary Figure S3) SIRT1 knockdown decreased LC3II expression and increased P62 expression, but Rab7 knockdown significantly increased LC3II and P62 expression, suggesting that Rab7 may stimulate the fusion of autophagic vacuoles. To elucidate the effect of SIRT1 and Rab7 on autophagy flux, we performed immunofluorescence staining on PANC-1 cells transfected with Ad-tf-LC3. SIRT1 knockdown significantly decreased the number of autolysosomes, indicated by the free red puncta, compared with the NC group. Rab7 knockdown increased the autophagosomes, indicated by yellow puncta, but decreased the red puncta compared with the NC group (Figure 5F).

### miR-138-5p inhibits autophagy and tumor growth *in vivo*

**Figure 6:**
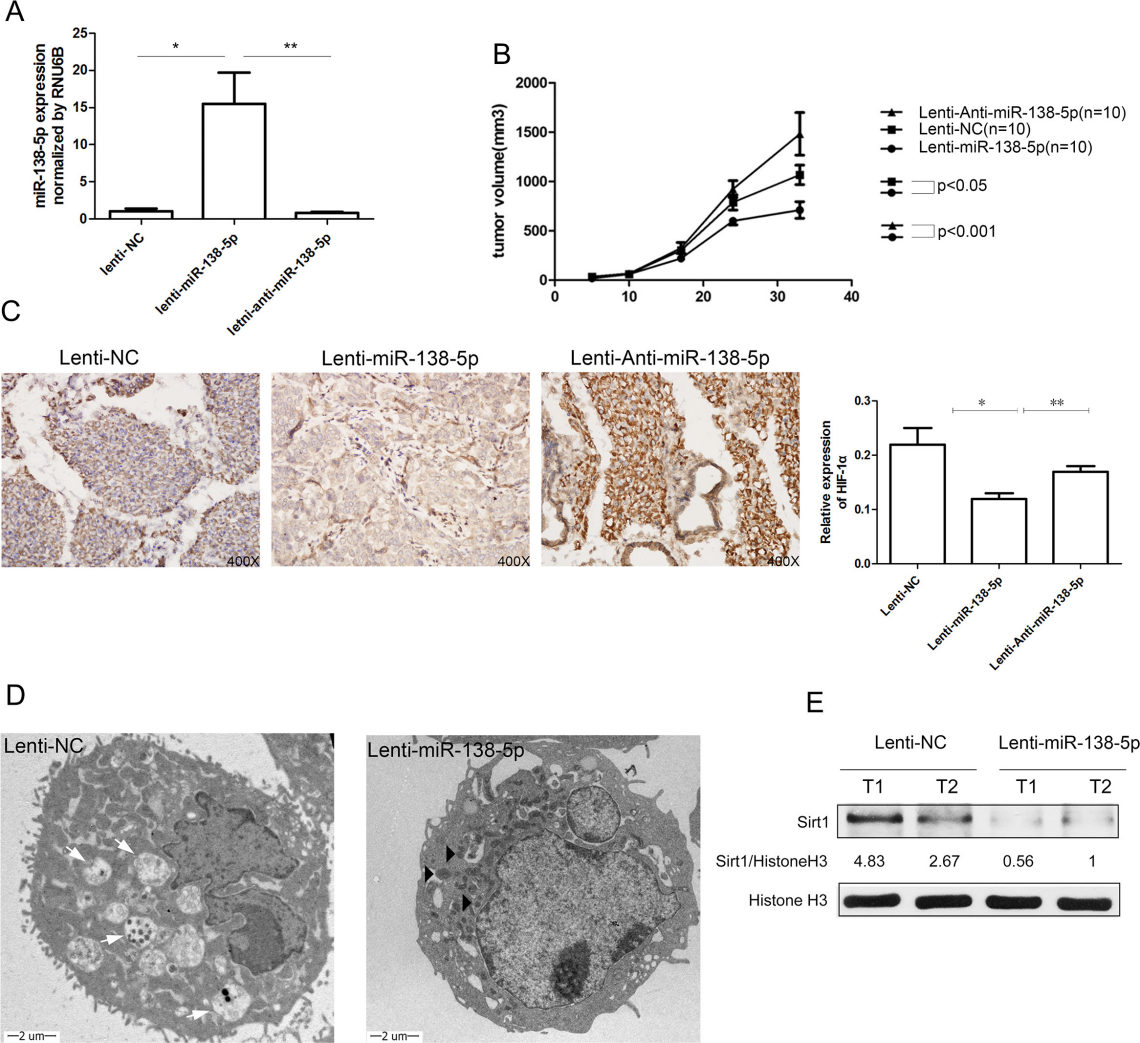
miR-138-5p inhibits autophagy and tumor growth of pancreatic cancer xenografts *in vivo* (**A**) Tumor growth curves are presented. (**B**) The expression of miR-138-5p was verified by quantitative PCR. (**C**) HIF-1α expression was measured by immunohistochemistry in xenograft tissues of each group and semi-quantitatively analyzed. (**D**) TEM showed the ultramicrostructure of representative xenograft tissue samples. White arrows in the Lenti-NC group indicate autophagosomes and autophagosome-fused lysosomes. Black triangles in the Lenti-miR-138-5p group indicate swollen mitochondria. (**E**) SIRT1 in two representative xenograft tissues in each group were detected by western blot analysis.

To evaluate whether miR-138-5p inhibits autophagy and tumor growth *in vivo*, we established stable miR-138-5p and anti-miR-138-5p cell lines by lentiviral transduction (Lenti-miR-138-5p, Lenti-anti-miR-138-5p). Lenti-NC was used as a control vector. Overexpression of miR-138-5p *in vivo* was confirmed by quantitative PCR (Figure 6A), and the increased miR-138-5p clearly retarded the growth of the subcutaneous tumors in nude mice (Figure 6B). HIF-1α was generally activated in subcutaneous tumors, suggesting that the tumor cells suffered from hypoxia. However, overexpressing miR-138-5p suppressed the expression of HIF-1α compared with the other groups (Figure 6C). TEM showed that Lenti-NC group had more autophagosomes and autophagosome-fused lysosomes, while the Lenti-miR-138-5p group had more swollen mitochondria (Figure 6D). Western blot analysis showed that SIRT1 was obviously decreased in the Lenti-miR-138-5p tumors, implying SIRT1 inhibition (Figure 6E).

## DISCUSSION

Modulating apoptosis has been shown to be a potential strategy for anti-cancer therapy over the past several decades. Promoting apoptosis is an approach whereby compromised cells are induced to commit suicide. It has been reported that autophagy has a critical role in tumor cell survival by maintaining energy production, leading to tumor growth and therapeutic resistance. The molecular mechanisms responsible for the autophagy-induced tumor survival have not been fully revealed. Furthermore, increasing evidence has pointed out that dysregulated miRNAs have an important role in autophagy regulation [17–20]. In this study, we screened a miRNA microarray of pancreatic cancer cells cultured under normoxic and hypoxic conditions and identified miR138-5p as a novel regulator of autophagy in pancreatic cancer cells. Hypoxia and nutrition starvation are central features of pancreatic cancer cells that can induce autophagy [21, 22]. Here, we found that miR-138-5p inhibits autophagy by directly targeting the autophagy-related gene *SIRT1* and that miR-138-5p directly binds to the 3′-UTR region of *SIRT1*. Knockdown of SIRT1 closely mimicked the autophagy-inhibiting effect of miR-138-5p overexpression in our cell models, although other unidentified targets may also be involved. These findings illustrate that SIRT1 is a key molecule regulated by miR-138-5p. Our results demonstrated that SIRT1 translocated into the nucleus and was downregulated in the cytoplasm compared with the matched normal pancreas tissue. It is possible that SIRT1 downregulation facilitates tumor cells to undergo mitophagy, which may be a protective response to survive metabolic stresses. Previously, we have shown that miR-138-5p can suppress pancreatic cancer cell proliferation.

In the current study, we found that downregulation of miR-138-5p was associated with induced autophagy in human pancreatic cancer tissues; however, restored expression of miR-138-5p inhibited the SIRT1/FoxO1/Rab7 pathway-dependent autophagy and subsequent apoptotic cell death. It was interesting to find that SIRT1 may repress pancreatic cancer cell development via multiple mechanisms, which makes miR-138-5p a potential target for tumor therapy. SIRT1 has been recognized as a nuclear protein that translocates into the cytoplasm in response to oxygen-glucose deprivation, resulting in increased sensitivity to apoptosis [23]. Previous studies have also demonstrated that SIRT1 is overexpressed in some cancers and correlates with poor prognosis due to its promotion of tumor metastasis. NAD^+^ as a receiver (SIRT1) of the acetyl group facilitates such cellular processes [24]. Further study has revealed that SIRT1 protein expression was reduced after vascular injury in mice [25]. SIRT1 has recently been reported to play an essential role in autophagy [26]. These findings indicate that cancer cells may require enhanced NAD^+^ synthesis and SIRT1 activity for survival and proliferation. However, our study showed that SIRT1 was decreased in the pancreatic cancer cell cytoplasm and mostly localized to the nucleus. SIRT1 translocated into the cytoplasm in response to nutrition deprivation as shown by western blot analysis. These data suggest that different subcellular populations of SIRT1 may have different roles in modulating cell apoptosis. Further study has revealed that SIRT1 could enhance the expression of deacetylated FoxO1 during nutrition starvation. Here, downregulation of SIRT1 decreased autolysosomes and inhibited autophagy, an effect very similar to that of miR-138-5p. Our research indicates that SIRT1 and FoxO1 may synergistically mediate autophagy under metabolic stress by forming a functional complex in pancreatic cancer cells. Deacetylation of FoxO1 induces the expression of genes involved in autophagy, including Rab7, which in turn stimulate autophagy. Rab7 may act as a docking, fusion, and autophagosome cluster [27]. Our results suggest that Rab7 plays an important role in the SIRT1/FoxO1 pathway of autophagy (Supplementary Figure S4). As Rab7 expression is downregulated and mature autophagic vacuoles fuse with the lysosome, we speculate that Rab7 may primarily contribute to the late stages of autophagy and the overall increase in autophagy flux. Sample size is a limitation of our research and only 29 patients are enrolled. We should enlarge the sample size and obtain patients’ survival time. In summary, our study, based on clinical samples, and cell and mouse models, highlights the role of miR-138-5p in autophagy modulation in pancreatic cancer cells.

## MATERIALS AND METHODS

### Ethical statement

This study was approved by the Human Research Ethics Committees at the Tongji Hospital, Tongji Medical College, HUST, and was carried out in accordance with the principles embodied in the Declaration of Helsinki.

All animal experiments were approved by the Committee on the Ethics of Animal Experiments of HUST (Permit no. 2016-S014). All treatments were carried out according to The U.S. Public Health Service Policy on the Humane Care and Use of Laboratory Animals. All surgical procedures were conducted under sodium pentobarbital anesthesia, and every effort was made to minimize animal suffering.

### Cell culture

The AsPC-1, BxPC-3, and PANC-1 cell lines were purchased from ATCC (Manassas, VA, USA). All cell lines were previously authenticated by ATCC with short tandem repeat (STR) typing. PANC-1 cells were grown in DMEM medium (Gibco, NY, USA); AsPC-1 and BxPC-3 cells were grown in RPMI-1640 medium (Gibco, NY, USA) supplemented with 10% fetal bovine serum (Gibco), 100 U/mL penicillin G, and 100 μg/mL streptomycin (Sigma, St. Louis, MO, USA) at 37°C in a humidified atmosphere containing 5% CO_2_.

### Autophagy induction

In order to induce autophagy, cells were incubated in a hypoxia incubator (Thermo Fisher Scientific, Heracell^™^ 240i, MA, USA) with a 1:5:94 mixture of O2/CO2/N2. In the nutrient starvation model, cells were washed three times with prewarmed PBS then incubated in EBSS medium (Sigma, NY, USA) at 37°C for 4 hr under 5% CO2.

### Quantitative real-time PCR (qPCR) analysis of miR-138-5p expression

qPCR analysis of miR-138-5p expression was performed on a LightCycler 480 (Roche Life Science, Indianapolis, IN, USA) and Agilent 2100 Bioanalyzer (Agilent Technologies, Santa Clara, CA, USA) using a TaqMan miRNA Assay according to manufacturer's protocol (Applied Biosystems, Foster City, CA, USA). All reactions were run in triplicate. miR-138-5p expression was normalized to the expression level of the housekeeping gene *U6*.

### RNA microarrays

Total RNA extracted from three normoxia cultured samples and three hypoxia-cultured samples was screened for differentially expressed genes by an Agilent RNA 6000 Nano Kit (Agilent Technologies). Primeview Human GeneChip (Agilent) was used for the analysis. RNA labeling and hybridization to Agilent miRNA microarray chips were performed with a GeneChip Hybridization Wash and Stain Kit (Agilent Technologies). Microarray data were deposited in the NCBI Gene Expression Omnibus public database (http://www.ncbi.nlm.nih.gov/geo/).

### miRNA-related reagents, small interfering RNAs, and transfections

miRNA precursors, inhibitors, and negative controls were purchased from Ambion (Thermo Fisher Scientific, MA, USA). *SIRT1* and *Rab7* small interfering RNAs (siRNAs) were purchased from RiboBio (Guangzhou, China). Transfections with 50 nM miRNA and 100 nM siRNA were performed using Lipofectamine RNAiMAX (Invitrogen, NY, USA) according to the manufacturer's instructions.

### miR-138-5p reagents and transfection

miR-138-5p mimic, miR-138-5p inhibitor, and NC were purchased from ThermoFisher Scientific (MA, USA). Human miR-138-5p overexpression adenoviruses was purchased from Vigene Biosciences (Shangdong, China); human miR-138-5p overexpression and knockdown lentiviruses, and negative control lentiviruses were purchased from Genechem (Shanghai, China). All transfections were carried out according to the manufacturer's instructions.

### Immunohistochemistry (IHC) and *in situ* hybridization(ISH)

For IHC analysis of SIRT1, 4 μm paraffin-embedded tissue microarray sections were incubated overnight at 4°C with primary antibodies against human SIRT1 (13161-1-AP, proteintech; 1:50 dilution). Sections were then washed and incubated for 30 min with biotinylated goat anti-rabbit IgG, washed thoroughly, and stained with diaminobenzidine. For ISH analysis of miR-138-5p, paraffin-embedded tissue sections were treated with miRCURY LNAtm Detection probe (Exiqon, Vedbaek, Denmark), following the manufacturer's protocol. Images were captured using an EnVision+ detection system (Dako, Wuhan, China). Two pathologists blinded to the patient's diagnosis and outcome examined each sample. Staining intensity and the proportion of positive cells were recorded.

### Western blot analysis

Proteins from human primary pancreatic cancer cells or subcutaneous xenografts were separated by electrophoresis and transferred to polyvinylidene difluoride membranes. Membranes were then incubated with diluted antibodies against LC3A/B [12741, Cell Signaling Technology (CST), Danvers, MA, USA], Atg3 (3415,CST), Atg5 (12994, CST), Atg7 (8558, CST), mTOR (cat.2983, CST), SQSTM1/p62 (8025, CST), FoxO1 (2880, CST), Rab7 (9367, CST), Ac-FoxO1/Ac-FKHR (sc-49437, Santa Cruz, CA, USA), caspase-3 (14220, CST), phospho-mTOR (5536, CST), SIRT1 (8469, CST), β-actin (60008-1-Ig, proteintech, Rosemont, IL, USA) or GAPDH (10494-1-AP, proteintech). Goat anti-rabbit and rabbit anti-mouse secondary antibodies were purchased from Boster (Wuhan, China). All antibody dilutions were 1:1000, except where otherwise indicated. After washing, blots were incubated with peroxidase-conjugated goat anti-rabbit IgG secondary antibody (CST; 1:2,000) and visualization by enhanced chemiluminescence (Boster).

### Immunoprecipitation assay

Whole-cell and freshly isolated glomeruli lysate proteins were used for immunoprecipitation (IP) with SIRT1 or FoxO1 antibody. Protein A/G-agarose beads were added, and the incubation was continued at 4°C overnight. Immunoprecipitates were extensively washed with lysis buffer and eluted with SDS loading buffer by boiling for 5 min. The bands were detected using the ChemiDoc XRS System (Bio-Rad, Hercules, CA, USA). The relative intensity of each band was normalized to β-actin.

### Apoptosis detection by annexin V-FITC staining

PANC-1 cells were not transfected or transfected with the indicated RNA duplexes for 48 h, and then incubated in serum-free Earle's balanced salt solution (EBSS) for another 24 h. Cells were then collected, washed twice with cold 1 × PBS and resuspended in 100 μL binding buffer, followed by incubation with Annexin V-FITC (BioVision, Milpitas, CA, USA) and propidium iodide (PI, Sigma-Aldrich) for 15 min at room temperature in the dark. Next, 200 μL of binding buffer was added, and the cells were analyzed by flow cytometry (Gallios, Beckman Coulter, Fullerton, CA, USA). Cells were considered to undergo apoptosis if they were Annexin V+/PI− (early stage of apoptosis) or Annexin V+/PI+ (end stage of apoptosis).

### Mitochondrial membrane potential assay by JC-1

Mitochondrial membrane potential (MMP) was analyzed using a Mitochondria staining kit (Cat No: C2006, Beyotime Biotechnology, Beijing, China). PANC-1 cells (5 × 10^5^ cells) were seeded in 6-well plates and incubated for 24 h in serum-free Earle's balanced salt solution (EBSS). Supernatants were removed from culture dishes and adherent cells detached with trypsin-EDTA. Cells were collected and resuspended using staining solution including 200× JC-1 at 1× staining buffer and incubated at 37°C in a CO2 incubator for 20 min. The stained cells were collected by centrifugation and washed once with 1 × JC-1 staining buffer. After one wash, the cell suspension was centrifuged once more and then the cells were resuspended in 1 ml staining buffer. Fluorescence intensity was analyzed using a flow cytometry (Gallios, Beckman Coulter, Fullerton, CA, USA).

### Adenoviruses and lentiviruses

Adenoviruses harboring miR-138-2 (Ad-miR-138-5p) mimics and its non-specific control were synthesized by Vigene (Vigene Biosciences, Shandong, China). Tandem fluorescent mRFP-GFP-LC3 was purchased from HANbio (HanBio, Shanghai, China). Recombinant lentiviruses harboring miR-138-5p, miR-138-5p-inhibitor or control lentivirus were prepared by GeneChem (Shanghai, China).

### Confocal microscopy and electron microscopy

PANC-1 cells grown on coverslips were fixed in 4% paraformaldehyde at room temperature for 15 min. Images were acquired using a confocal laser imaging system (TCS SP5, Leica, Guizhou, China). The average number of LC3 puncta per cell was quantified. At least 50 cells were evaluated for each sample. For electron microscopy, cells or fresh tissues were fixed in 2.5% glutaraldehyde in 0.1M cacodylate buffer at 4°C overnight, and then post-fixed in phosphate buffer with 1% osmium tetroxide for 1 h. After dehydration in a graded series of acetone, the cells were infiltrated and embedded in spur resin. Sectioned grids were stained with 2% uranyl acetate in 50% methanol for 10 min, followed by 1% lead citrate for 7 min, and imaged by a Hitachi H-7000FA transmission electron microscopy (TEM).

### Luciferase reporter assay

PANC-1 cells grown in a 24-well plate were co-transfected with 5 nM of NC or miR-138-5p duplex, and 10 ng of Dual-luciferase reporter plasmid (Riobio, Guangzhou, China), which comprises pmiR-REPORT control vector, Luc-SIRT1, or Luc-SIRT1-mu vectors. The luciferase assay was carried out following the manufacturer's instructions. The assay was performed at least three times in independent experiments.

### Tissue microarrays

Tissue microarrays containing pancreatic cancer tissue samples (HPan-Ade060CS-01) were obtained from Shanghai Outdo Biotech (Shanghai, China). Surgical specimens of pancreatic cancers and corresponding normal pancreas tissues were obtained from 29 patients. Another 2 pancreas tissues obtained from 2 bile duct tumor patients were taken as control. All tissues were resected from January 2015 to March 2016. The 29 patients with pancreatic cancer encompassed 21 males and 8 females of mean age 60.92 years (range, 35–84 years). Histologically, all 29 cancers were adenocarcinomas. Patients did not receive preoperative radiotherapy or chemotherapy. More detailed information can be found in Supplementary Table S2.

### *In vivo* animal experiments

BALB/c nude mice (female; 4 weeks old) were obtained from HFK BIOSCIENCE (Beijing, China). The mice were randomly divided into three groups (negative control lentivirus group, *n* = 10; miR138-5p overexpressed lentivirus group, *n* = 10; miR138-5p downregulated lentivirus group, *n* = 10). PANC-1 cells (3 × 10^6^) in 100 μl of PBS were inoculated subcutaneously into a female BALB/c athymic nude mouse at 6 weeks of age. The tumor volume (V) was recorded by measuring the length (L) and width (W) with a vernier caliper, and calculated with the formula V = (L × W^2^) × 0.5. Mice were sacrificed 6 weeks after inoculation.

### Statistical analyses

The results of continuous variables are presented as mean ± SD unless otherwise stated. Treatment groups were compared using independent sample *t*-tests. Pair-wise multiple comparisons were performed by one-way ANOVA (two-sided). *P* < 0.05 was considered statistically significant. All analyses were performed using IBM SPSS Statistics software version 17.0 (Chicago, IL, USA).

## SUPPLEMENTARY MATERIALS FIGURES AND TABLES





## Abbreviations

ATG3: autophagy related 3
ATG5: autophagy related 5
ATG7: autophagy related 7
FoxO1: forkhead box O1
SIRT1: sirtuin 1
Rab7: member RAS oncogene family
HUST: Huazhong University of Science and Technology
