# Supplementary material for: miR-138-5p suppresses autophagy in pancreatic cancer by targeting SIRT1
Source: Oncotarget. 2016 Dec 29;8(7):11071–82. doi: 10.18632/oncotarget.14360 (PMC5355247; doi:10.18632/oncotarget.14360)
Supplement: Supplementary file 1 [file oncotarget-08-11071-s001.pdf]

miR-138-5p suppresses autophagy in pancreatic cancer by targeting *SIRT1*

Supplementary Materials

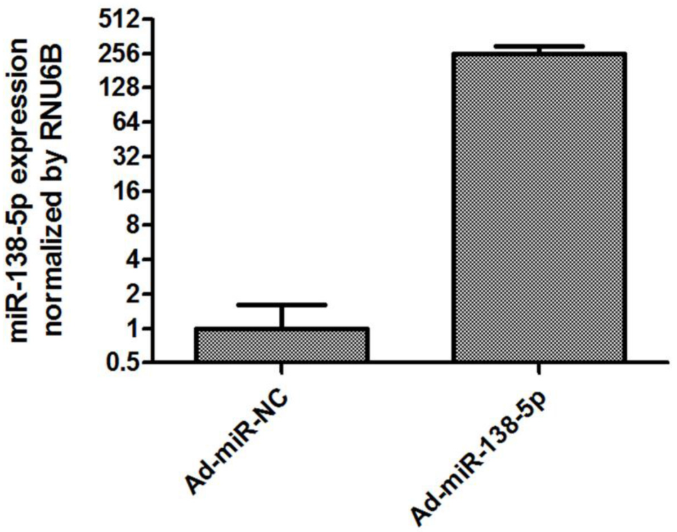

Supplementary Figure S1: Indicated cells were subjected to PCR to detect miR-138-5p expression.

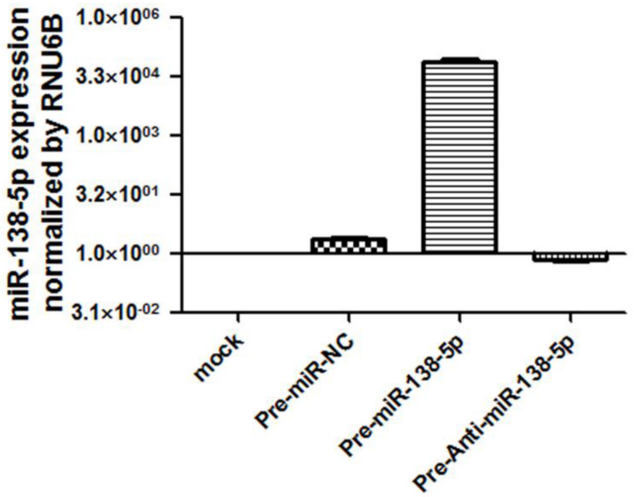

Supplementary Figure S2: Indicated cells were subjected to PCR to detect miR-138-5p expression.

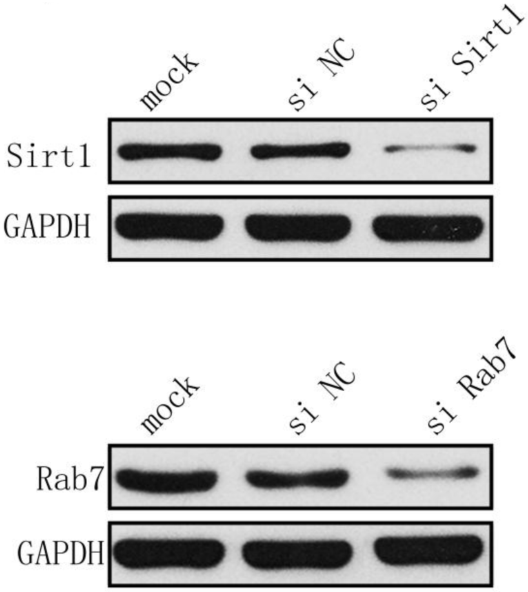

Supplementary Figure S3: Indicated cells were subjected to western blot to detect SIRT1 and Rab7 expression.

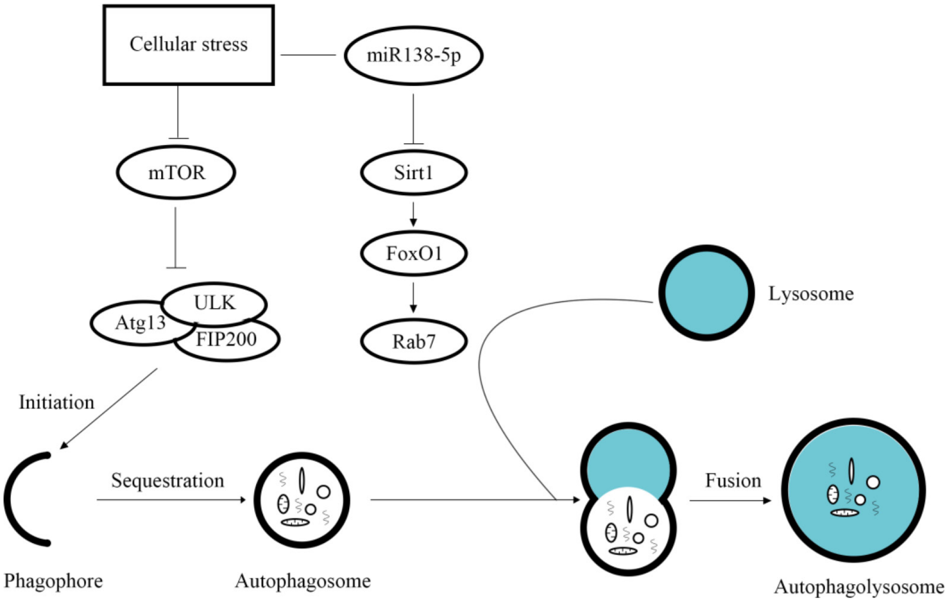

Supplementary Figure S4: Schematic illustration for the role of miR-138-5p on autophagy in pancreatic cancer. miR-138-5p inactivates SIRT1 through binding its 3′-UTR site. Inactivation of SIRT1 promote apoptosis probably through SIRT1-FoxO1-Rab7 axis.

Supplementary Table S1: Association between SIRT1 expression and clinicopathological features of pancreatic caner

	Patients	SIRT1 expression		*P* value
		Low	High	
**Gender**				
Male	21	12	9	0.122
Female	8	5	3	
**Age(Year)**				0.244
< 65	15	7	8	
≥ 65	14	11	3	
**Tumor size(cm)**				0.550
≤ 3	14	9	5	
> 3	15	6	9	
**Tumor stage**				0.228
CIS, Ta, T1	6	1	5	
T2–T4	23	10	13	
**Tumor grade**				0.023*
Grade I, II	22	15	7	
Grade III	7	1	6	
**Lymphnodes status**				0.278
Negative	21	13	8	
Positive	8	6	2	
**Surrounding tissues infiltration**				0.023*
Negative	12	5	7	
Positive	17	14	3	

*$p < 0.05$.

Supplementary Table S2: The detailed information of the patients enrolled in tissue array. See Supplementary_Table_S2
