# Supplementary material for: miR-138-5p suppresses autophagy in pancreatic cancer by targeting SIRT1
Source: Oncotarget. 2016 Dec 29;8(7):11071–82. doi: 10.18632/oncotarget.14360 (PMC5355247; doi:10.18632/oncotarget.14360)
Supplement: Supplementary file 2 [file oncotarget-08-11071-s002.docx]

**Supplementary Table S2:** **The detailed information of the patients enrolled in tissue array**

| Patient number | diagnosis | surgery | gender | age | Date of operation | Chief Complaint | distant metastasis | Primary  organ | Pathological type | Pathological grading | Tumor size | Total number of lymph nodes | Positive number | T | N | M |  |
| --- | --- | --- | --- | --- | --- | --- | --- | --- | --- | --- | --- | --- | --- | --- | --- | --- | --- |
| 1 | Pancreatic ductal adenocarcinoma | PD | F | 68 | 2015/1/16 | Epigastric discomfort with jaundice more than 1 month | no | pancreas | ductal adenocarcinoma | Ⅱ | 2×2×1.5cm | 16 | 0 | T1 | N0 | M0 |  |
| 2 | Pancreatic ductal adenocarcinoma | PD | M | 48 | 2015/2/6 | Repeated upper abdominal pain with intermittent reflux than 3 years | no | pancreas | ductal adenocarcinoma | Ⅰ-Ⅱ | 1.6×1.5×1cm | 16 | 0 | T1 | N0 | M0 |  |
| 3 | Pancreatic ductal adenocarcinoma | PD | F | 66 | 2015/2/28 | Abdominal pain and discomfort more than 1 month | no | pancreas | ductal adenocarcinoma | Ⅱ | 2×2×1.5cm | 4 | 0 | T1 | N0 | M0 |  |
| 4 | Pancreatic ductal adenocarcinoma | PD | M | 60 | 2015/3/10 | Epigastric discomfort with jaundice more than 2 weeks | no | pancreas | ductal adenocarcinoma | Ⅱ | 2×2×2cm | 8 | 0 | T1 | N0 | M0 |  |
| 5 | Pancreatic ductal adenocarcinoma | PD | M | 84 | 2015/4/1 | Upper abdominal pain and discomfort more than 3 month | no | pancreas | ductal adenocarcinoma | Ⅰ-Ⅲ | 2×1.5×1.5cm | 9 | 0 | T1 | N0 | M0 |  |
| 6 | Pancreatic ductal adenocarcinoma | PD | M | 70 | 2015/4/7 | Abdominal pain and discomfort more than 1 year | no | pancreas | ductal adenocarcinoma | Ⅱ | 2×2×2cm | 10 | 0 | T1 | N0 | M0 |  |
| 7 | Pancreatic ductal adenocarcinoma | PD | M | 67 | 2015/4/15 | Obstructive jaundice 10 days | no | pancreas | ductal adenocarcinoma | Ⅱ | 6×6×5cm | 8 | 0 | T2 | N0 | M0 |  |
| 8 | Pancreatic ductal adenocarcinoma | PD | M | 53 | 2015/6/4 | Skin and sclera yellow dye 7 days | no | pancreas | ductal adenocarcinoma | Ⅲ | 3×2.5×2.5cm | 4 | 0 | T2 | N0 | M0 | |
| 9 | Pancreatic ductal adenocarcinoma | PD | M | 64 | 2015/7/8 | Abdomen mass 3 weeks | no | pancreas | ductal adenocarcinoma | Ⅱ | 3×2.5×2cm | 2 | 0 | T2 | N0 | M0 |  |
| 10 | Pancreatic ductal adenocarcinoma | PD | M | 51 | 2015/7/16 | Upper abdominal pain and Obstructive jaundice 3 days | no | pancreas | ductal adenocarcinoma | Ⅰ-Ⅱ | 4×3×3cm | 20 | 0 | T2 | N0 | M0 |  |
| 11 | Pancreatic ductal adenocarcinoma | PD | M | 70 | 2015/7/21 | Skin and sclera yellow dye more than 2 weeks | no | pancreas | ductal adenocarcinoma | Ⅱ | 3×3×2cm | 7 | 0 | T2 | N0 | M0 |  |
| 12 | Pancreatic ductal adenocarcinoma | PD | M | 69 | 2015/9/8 | Abdominal pain and discomfort more than 2 weeks | no | pancreas | ductal adenocarcinoma | Ⅱ | 5.5×3×3cm | 14 | 0 | T2 | N0 | M0 |  |
| 13 | Pancreatic ductal adenocarcinoma | PD | F | 36 | 2015/9/16 | Upper abdominal pain for 3 years | no | pancreas | ductal adenocarcinoma | Ⅱ | 4×2×2cm | 5 | 0 | T2 | N0 | M0 |  |
| 14 | Pancreatic ductal adenocarcinoma | PD | M | 72 | 2015/9/21 | Abdominal pain and discomfort more than 4 months | no | pancreas | ductal adenocarcinoma | Ⅱ-Ⅲ | 4×3×2cm | 9 | 0 | T3 | N0 | M0 |  |
| 15 | Pancreatic ductal adenocarcinoma | PD | F | 69 | 2015/9/23 | Epigastric discomfort for 6 months,with Skin and sclera yellow dye 4 days | no | pancreas | ductal adenocarcinoma | Ⅱ | 3×3×2.5cm | 11 | 0 | T3 | N0 | M0 |  |
| 16 | Pancreatic ductal adenocarcinoma | PD | M | 68 | 2015/10/8 | Hemafecia for 3 months | no | pancreas | ductal adenocarcinoma | Ⅱ | 4×4×4cm | 2 | 0 | T3 | N0 | M0 |  |
| 17 | Pancreatic ductal adenocarcinoma | PD | M | 66 | 2015/10/15 | Skin and sclera yellow dye 10 days | no | pancreas | ductal adenocarcinoma | Ⅱ | 2×1×1cm | 10 | 0 | T3 | N0 | M0 |  |
| 18 | Pancreatic ductal adenocarcinoma | PD | M | 35 | 2015/10/27 | Epigastric discomfort for 6 months,with backache 3 months | no | pancreas | ductal adenocarcinoma | Ⅱ | 5×4×4cm | 5 | 2 | T2 | N1 | M0 |  |
| 19 | Pancreatic ductal adenocarcinoma | PD | F | 52 | 2015/10/28 | Upper abdominal pain for 1 years | no | pancreas | ductal adenocarcinoma | Ⅱ | 6×3×3cm | 12 | 3 | T2 | N1 | M0 |  |
| 20 | Pancreatic ductal adenocarcinoma | PD | M | 60 | 2015/11/3 | Abdominal pain and discomfort more than 6 months | no | pancreas | ductal adenocarcinoma | Ⅱ | 3×3×2cm | 3 | 1 | T2 | N1 | M0 |  |
| 21 | Pancreatic ductal adenocarcinoma | PD | M | 71 | 2015/11/13 | Epigastric discomfort for 3 months | no | pancreas | ductal adenocarcinoma | Ⅲ | 6×5×4cm | 10 | 4 | T2 | N1 | M0 |  |
| 22 | Pancreatic ductal adenocarcinoma | PD | F | 62 | 2015/11/24 | Epigastric discomfort for 9 months | no | pancreas | adenosquamous carcinoma | Ⅱ-Ⅲ | 6×6×4cm | 1 | 1 | T3 | N1 | M0 |  |
| 23 | Pancreatic ductal adenocarcinoma | PD | M | 58 | 2015/12/7 | Skin and sclera yellow dye more than 2 weeks | no | pancreas | ductal adenocarcinoma | Ⅱ | 3.8×3×2.5cm | 12 | 1 | T3 | N1 | M0 |  |
| 24 | Pancreatic ductal adenocarcinoma | PD | M | 68 | 2015/12/15 | Epigastric discomfort for 2 months | liver | pancreas | ductal adenocarcinoma | Ⅱ | 9×4×4cm | 3 | 0 | T2 | N0 | M1 |  |
| 25 | Pancreatic ductal adenocarcinoma | PD | M | 62 | 2015/12/22 | Epigastric discomfort for 3 years | liver | pancreas | ductal adenocarcinoma | Ⅱ | 5×4×3cm | 4 | 0 | T2 | N0 | M1 |  |
| 26 | Pancreatic ductal adenocarcinoma | PD | F | 60 | 2016/1/25 | Upepigastric and back dis comfort for 10 months | liver | pancreas | ductal adenocarcinoma | Ⅱ | 3.5×3×3cm | 12 | 2 | T3 | N1 | M1 |  |
| 27 | Pancreatic ductal adenocarcinoma | PD | M | 53 | 2016/1/28 | Left epigastric discomfort for 3 months，be aggravated 1 week | paranephros | pancreas | adenosquamous carcinoma | Ⅱ-Ⅲ | 5×4.5×4cm | 4 | 0 | T3 | N0 | M1 |  |
| 28 | Pancreatic ductal adenocarcinoma | PD | M | 60 | 2016/2/24 | Epigastric discomfort for 2 month, the skin and sclera yellow dye more than1 month | paranephros | pancreas | ductal adenocarcinoma | Ⅱ | 3×3×2cm | 12 | 4 | T3 | N1 | M1 |  |
| 29 | Pancreatic ductal adenocarcinoma | PD | F | 60 | 2016/3/15 | Skin and sclera yellow dye, urine color deepened for 2 weeks | spleen | pancreas | ductal adenocarcinoma | Ⅲ | 3×3×2.5cm | 4 | 0 | T2 | N0 | M1 |  |
| 30 | Distal bile duct cancer | PD | M | 32 | 2015/1/19 | Obstructive jaundice 1 weeks | no | biliary ducts | adenocarcinoma | I | 1×1×2.5cm | 6 | 0 | T1 | N0 | M0 |  |
| 31 | Distal bile duct cancer | PD | M | 39 | 2015/9/21 | Skin and sclera yellow dye more than 5 days | no | biliary ducts | adenocarcinoma | I | 1×2×2.5cm | 8 | 0 | T1 | N0 | M0 |  |
